# Greenhouse gas emissions as sustainability indicators in agricultural sectors’ adaptation to climate change: Policy implications

**DOI:** 10.4102/jamba.v11i1.576

**Published:** 2019-06-19

**Authors:** Tom E. Volenzo, John O. Odiyo, John Obiri

**Affiliations:** 1 Department of Hydrology and Water Resources, School of Environmental Sciences, University of Venda, Thohoyandou, South Africa; 2 School of Disaster Management and Sustainable Development, Masinde Muliro University of Science and Technology, Kakamega, Kenya

**Keywords:** climate change adaptation, disaster risk reduction, externalities, greenhouse gases, sustainability

## Abstract

Effective adaptation action to climate change requires a balance between reducing vulnerabilities and managing risks. However, in most adaptation actions, risks such as greenhouse gas emissions, and those that impose negative externalities on global communities and ecosystems, are often overlooked. This article contextualises adaptation of maize stover (MS) as a dairy cattle feed among resource-poor farmers in western Kenya. In so doing, it attempts to establish the nexus between resource constraint and maladaptation to climate change. Simulation of methane emissions was carried out from secondary data and a survey of dairy cattle feeding strategies by resource-poor farmers. The level of greenhouse gas emissions in dairy feeding strategies is used as a measure and indicator of sustainability. Using disaster risk reduction principles, policymakers and community of practice in climate change action are encouraged to design and implement policies and strategies that take cognisance of poverty–maladaptation–environmental degradation nexus.

## Introduction

Many parts of the world have been experiencing growing urbanisation and change in dietary preferences that favour dairy production. However, a current and projected increase in levels of milk production would not be possible without expanding production and yield of crop agriculture, hence an increase in demand for land. The lack of additional available land except in parts of tropical Latin America prohibits horizontal expansion of existing modes of dairy cattle production. This necessitates search for alternative dairy feed resources (Steinfeld, Wassenaar & Jutzi 2006). This is more so because of feed constraints that are imposed by climate change. Expanding dairy production in many parts of the world, as such, has serious implications on climate change risk management.

Worldwide, wild and domestic ruminants, as a result of metabolic processes (enteric fermentation), produce 15% – 25% of total methane gas emissions, 74% of which is caused by cattle (Tamminga 1996). Enteric fermentation generates approximately 86 million tonnes of CH_4_ worldwide (Steinfeld et al. 2006). Managing methane emissions from ruminants in general, and dairy cattle feeding strategies in particular, is important in climate change mitigation (Volenzo 2015). Mitigating greenhouse gas (GHG) emissions requires comprehensive action by policymakers, producers and consumers (Koneswaran & Nierenberg 2008).

Ruminants produce carbon (IV) oxide and methane (CH_4_). However, carbon (IV) oxide (CO_2_) produced by ruminants, notably cattle, is of less concern because it originates entirely from newly generated biomass and does not contribute to its net rise in the atmosphere. This leaves methane as the gas of concern (Tamminga 1996). Methane emissions increase climate forcing risks and vulnerability of resource-constrained households to other external shocks. Based on the warming potential, methane emission has higher social costs compared to CO_2_ (Hope 2008b). Methane gas emission has a warming potential 20 times greater than CO_2_ on gram per gram basis over a period of 100 years (Tamminga 1996).

Although there is high uncertainty in the models used to estimate social costs from emissions, the social cost of CH_4_ is estimated to grow 50% faster per year compared to 2.4% for carbon dioxide (Hope 2008a). This is attributed to shorter atmospheric lifetime of CH_4_ in comparison to CO_2_ (IPCC 1996). Dairy cattle feeding strategies are, as such, critical to climate change risk management. Methane emissions compound weather variability and climate change risks and the magnitude of global warming. This increases the vulnerability of agricultural based livelihoods to new anthropically induced disaster risks.

### Significance statement

The nexus approach to environmental resource management examines the inter-relatedness and interdependencies of environmental resources through the concept of trade-offs and synergies (Kurian & Meyer 2015). This underscores the need to investigate policies, plans and programmes in terms of risks and opportunities. Building upon this thinking, and the comprehensive conceptual model for disaster management (Asghar, Alahakoon & Churilov 2005), the concept of sustainable development using levels of GHGs emissions as sustainability indicators is explored. We adapt the comprehensive conceptual model for disaster management, which links strategic planning to hazard assessment, risk management, disaster management actions (mitigation, preparedness, response and recovery) and environmental conditions that impact the severity of a disaster in making inferences.

By investigating risk attitude and risk management among resource-poor small-scale dairy farmers, we are able to link risk attitude and livelihood strategies in adaptation to climate to livelihoods outcomes and GHGs emission levels, hence the poverty–production risk–maladaptation–environmental degradation nexus. The main contribution of this article is thus a robust analytical framework that integrates socio-ecological interfaces in adaptation planning and nexus thinking. The framework makes it possible to analyse climate adaptation-related risks, as well as trade-offs and synergies in climate change action. It further informs policy on holistic and integrated approaches that could bridge mitigation–adaptation divide in climatic change action decision support systems, transdisciplinary and sustainable development agenda.

### Ethical considerations

Research authorisation for the study was obtained from the National Council for Science and Technology (reference number: NCST/RCD/10/013/23).

### Analytical framework

Unplanned or autonomous adaptation to climate change could be a driver to degradation of land resources, ecosystems and biodiversity, with far-reaching negative impacts on food security, incomes of small-scale farmers and poverty reduction initiatives. According to Jung et al. (2012), development paths and the choices that define adaptation may affect the severity of climate impacts, not only through changes in exposure and sensitivity but also through changes in the capacities of systems to adapt. This includes local-scale disaster risk reduction (DRR) and resource management and broader social dimensions (Haddad 2005). However, under generic adaptation planning, uncertainties remain regarding how effective global actions will be in reducing GHGs (Banuri & Opschoor 2007). Anchoring adaptation planning on evidence and verifiable knowledge can reduce such uncertainties and increase the effectiveness of mitigation measures.

According to C-CIARN Agriculture (2004), adaptation to climate and weather variability risks should not only take advantage of opportunities, but also increase resilience of farmers’ production systems. This implies minimising environmental degradation and/or pollution risks, as well as stabilisation of output and income. In tandem with sustainability paradigm, anticipatory adaptation implies identifying and reducing underlying risk factors associated with development policies, plans and programmes and linking climate change adaptation to DRR in a mutually supportive manner (UNISDR 2015). Climate change adaptation policies in the agricultural sector should, therefore, screen for and evaluate the economic, social and environmental costs (externalities) against potential benefits.

To maintain or improve their livelihoods, farmers have to adapt to changing policy contexts and environment in which they operate (Maredia & Minde 2002). The challenging task in planning adaptation activities involves finding ways to combine different measures in a meaningful way in order to avoid maladaptation. The most attractive adaptation measures are those that offer benefits in the near future as well as reduce vulnerabilities in the long-term (Mimura et al. 2014). This increases the need to investigate policies, plans and programmes in terms of risks and opportunities. The role of adaptive capacity, adaptation process and their implication on use of GHG emissions as a sustainability indicator is particularly relevant in this context.

Given that disasters are potentially embedded in implementation of socio-economic policies and interventions formulated to manage climate change, it is posited that failure to identify, quantify and treat risks embedded in dairy feeding adaptation initiatives actually enhances climate change risks and exacerbates small-scale farmers’ vulnerability to climate change and weather variability (Volenzo 2015). Adaptation may result in suboptimal outcomes and unintended adverse impacts for other sectors.

Maladaptation occurs when adaptation action or investment taken to avoid or reduce climate change impacts increases vulnerability to other adverse impacts or increases the vulnerability of other systems, sectors or social groups (Adger, Arnell & Tompkins 2005; Barnett & O’Neill 2010). Maladaptation imposes negative externalities on third parties and ecosystems. A good example of maladaptation would be suboptimal milk production levels, hence increased CH_4_ emissions per litre of milk from use of MS under low or non-existent energy and protein supplementation. The resultant negative externalities because of radiative forcing may have disproportionate impact on the poor by increasing their vulnerability to other external shocks.

The main objective in scaling up sustainable agriculture practices is to transform food production from a major GHG emitter to a net neutral and possibly a GHG sink (UNEP 2011). This can be achieved through socio-economic policies and regulatory instruments (UNEP 2012). Sustainable development goals (SDGs) commit subscribing nations to targets aimed at achieving sustainable water use, energy use as well as sustainable agricultural practices (UN 2015). Sustainable adaptation to climate change and climate policies in agriculture presuppose increased farm productivity and income, resilience to shocks and mitigation of GHGs (Asghar et al. 2005; UNEP 2011). Dairy feed supplementation is a risk management strategy, which in turn influence dairy cattle productivity and CH_4_ emission risks (Volenzo 2015).

Previous studies have postulated several relationships in the analysis of poverty–environmental degradation nexus. In one of the relationships, poverty–environmental degradation nexus is considered in a bidirectional order for policy making purposes, that is, the prioritisation of environmental management or poverty alleviation interventions. It was argued by Dasgupta et al. (2005) that irrespective of policy priorities, complementarity between environmental and poverty alleviation strategies should always be pursued. However, there is paucity of research findings on poverty–production risks–maladaptation to climate change nexus. Existing findings on poverty–environmental degradation nexus can be used in gaining insights into the proposed poverty–price–production risks–maladaptation to climate change nexus.

Table 1 provides simulated effect of supplementation on milk production and methane emission risks in maize stover (MS) feeding strategies. The results suggest that the use of MS as standalone ration increases methane emission risks by up to 30.9% per kilogram of un-supplemented MS (18.03 to 26.11 Mj [megajourles] kg^−1^). The higher marginal increment in CH_4_ emissions, on average, exacerbates radiative forcing than what would be under higher dairy productivity. Addressing the multiple risks faced by the smallholder dairy farmers can positively influence integration and intensification of dairy feeding strategies that use MS to reduce methane emission risks by 30% (Volenzo 2015). In addition, the lack of and/or suboptimal supplementation in dairy cattle feeding undermines participation of resource-poor farmers in better remunerative livelihoods and confines them into the poverty traps.

**TABLE 1:** Simulated effect of supplementation on milk and methane emission risks.

Ration	DMI gKg^−1^	CP (%)	ME (%)	Prod (L)	Kes/Kg ration	Kes/Kg/L	CH_4_ MjKg^−1^	CH_4_ MjKg^−1^/L
Ms	910.0	4.0	2.00	3	5.00	0.60	26.10	8.70
Ms+L	782.6	8.2	2.10	7	5.60	1.25	18.07	2.58
Ms+u	890.0	6.6	2.00	6	5.45	0.91	25.73	4.30
Ms+CSC+U	872.0	14.1	1.58	10	19.00	1.90	24.04	2.40
Ms+CSC+U+M	857.8	16.6	2.31	17	24.20	1.45	23.60	1.39
Napier	675.0	6.5	7.50	5	20.00	4.00	17.90	3.58
Napier+L	596.0	12.0	6.50	10	21.50	2.15	16.80	1.70
Napier+CSC	700.0	13.8	3.86	12	26.00	2.17	17.60	1.47
Napier+CSC+M	635.0	14.3	11.70	15	29.80	1.99	17.10	1.14
Ms+Napier	793.0	5.3	4.80	5	12.50	2.50	18.09	3.62
Ms+Napier+CSC	795.0	14.9	4.15	16	23.75	1.48	18.08	1.13
Ms+Napier+CSC+M	786.0	16.9	4.00	18	28.00	1.56	18.07	1.00

Note: Urea (CP of 265, Loosli and McDonald, 1968) given at maximum of 10 gKg^−1^ of ration. Ms/Napier ration in ratio of 1:1 while, CSC and legume fodder and molasses do not exceed 30% and 20% of the ration, respectively.

CSC, Cotton seed cake; M, molasses; Ms, maize stover; U, urea; L, legume fodder; ME, metabolisable energy Mcal/Kg; CP, crude protein; DMI, potential dry matter intake; Prod, production based on critical thresholds and literature data.

Volenzo (2015) provides some evidence on centrality of and linkages between farmers’ risk attitude and the adoption of environmentally sustainable technologies, dairy cattle productivity and CH_4_ emission. Because price and market risks are a function of institutional arrangements and policy contexts, Volenzo argues that policymakers may utilise risk management instruments in influencing dairy sector producers to mitigate climate change risks. This is on the account that short-term decisions about allocation of assets and livelihood adaptation options can have both long-term negative and positive implications on the environment and the cycle of poverty (Siegel 2005).

Modelling and simulation can be used in the assessment of poverty–production risks–maladaptation to climate change nexus. As simulated quantitative models offer a range of scenarios, they can be utilised in an iterative way to develop scenarios that project impacts within a coupled human–environment system (Turner et al. 2010). The worst-case scenario in the simulation model may be used in disaster planning (Downing & Patwardhan 2002). A model on methane emission in ruminants by Mills et al. (2003) was used in this study to simulate methane emissions from dairy cattle feeding strategies by resource-constrained farmers.

Vulnerabilities to climate change risks, various policy responses and economic actions (adaptation) at household and national levels, as well as resource base can be used to simulate outcomes of adaptive action. In dairy cattle production, energy and protein supplementation are a production risk management strategy. The analogy is applied in this article to assess the effect of MS supplementation on emission of methane.

**FIGURE 1:**
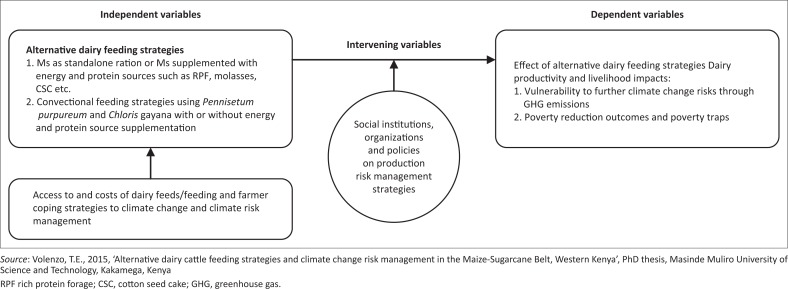
Conceptual framework.

From the above analysis, the authors suggest an analytical framework (Figure 1) for policymakers and the community of practice. The model illustrates the linkage between small-scale dairy farmers’ production risk management strategies and climate change risks. The model can be applied for the diagnosis, development, monitoring and evaluation of adaptation policies training and research programmes, as well as for the development of extension packages for the agricultural sector in general, and small-scale dairy farmers in particular.

### Contextualising the critical issues

The introductory and the analytical frameworks provide evidence on poverty–maladaptation–environmental degradation nexus. Therefore, this article focuses on specific issues that are critical to sustainability and climate change mitigation and adaptation and argues that integrating the risk component provides the basis for comprehensive policy analysis and response, risk assessment and mainstreaming of sustainability concerns into agricultural sector adaptation to climate change. Using the level of methane emission from different dairy feeding strategies, issues that impinge on poverty–maladaptation–environmental degradation nexus are examined.

## Maize–sugarcane belt, western Kenya

Changes in climate and their effects have serious threats on the stability and productivity of the agricultural sector (FAO 2010). A study on the economic impact of climate change threats revealed that future economic costs of the impact of climate change on market and non-market sectors (human health and environment) in Kenya might be close to 2.6% of gross domestic product (GDP) per year by 2030 and potentially greater than 50% by 2050 (SEI 2009).

The changing climatic conditions, particularly rainfall and temperature patterns, portend adverse impacts on Kenya’s socio-economic sectors, with current projections indicating that such impacts will worsen in the future if significant reductions in the anthropogenic GHGs emissions that are responsible for climate change are not made (GOK 2010). As agriculture plays a pivotal role in Kenya’s economy, moderating actual or potential climate change damages or adaptation to the climate change risks in a sustainable manner is critical.

About 40% of dairy cattle in Kenya are found in semi-intensive farming systems. This feature, coupled with information that such production systems have the highest maize densities, indicates a high potential for benefits from maize–livestock integration (Thorne et al. 2002). In view of the stresses occasioned by climate change and weather variability, maize–dairy integration offers an opportunity to manage production risks in a dairy enterprise. Unlike other fodder production systems, dry MS forage- based systems do not compete for limited food crop production niches (Volenzo 2015).

**FIGURE 2:**
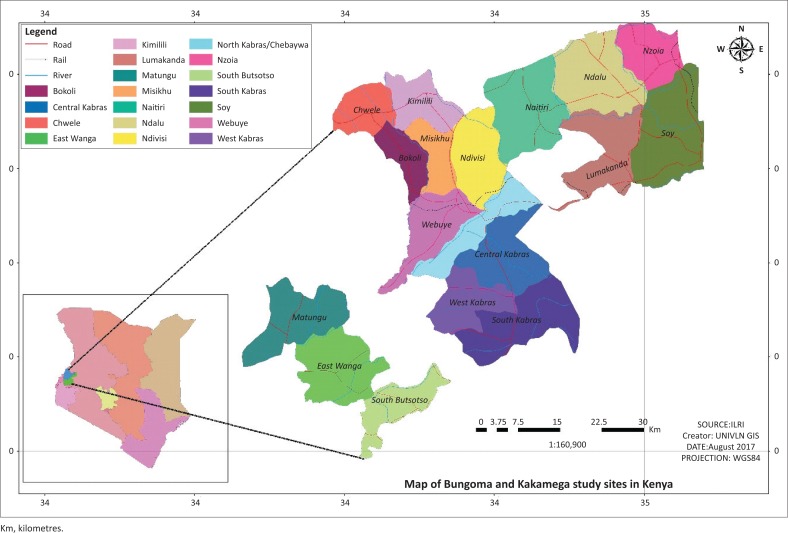
Map of study area (GIS generated).

The study was conducted in Bungoma and Kakamega counties located between longitude 34°25’E and 35°10’E and latitude 0°1’N and 0°15’ (Jaetzold et al. 2005). The area is characterised by commercial sugarcane farming as well as maize production at subsistence and commercial levels as major economic activities (KNBS 2009). Livestock–crop integration that serves a myriad of purposes (Ongadi et al. 2010) is a common characteristic in majority of the households. Figure 2 provides a map of the study area.

The western Kenya maize–sugar belt is under increased population pressure (Jaetzold et al. 2005). Diminishing land sizes and seasonality in production of the feeds makes it difficult to bridge protein and energy gaps in dairy cattle feeding (Ongadi et al. 2010). Climate change is expected to widen and compound difficulties in bridging energy and protein gaps. Such scenarios point towards increased vulnerability of dairy farmers to climate change and weather variability risks. Furthermore, high market risks faced by the farmers negatively impact the adoption of technologies that have potential for increased productivity, low net or GHG neutral emission risks as well as poverty alleviation (Volenzo 2015).

### Climate change

Climate change refers to the change in the state of climate whether due to natural variability or as a result of human activity that can be identified by changes in the mean and/or the variability of its properties, and that persist for extended period, typically decades or longer (IPCC 2007). Methane, nitrous oxide and carbon dioxide are the main GHGs that contribute to global warming and depletion of ozone layer (Steinfeld et al. 2006). Emission of GHGs traps heat in the atmosphere, leading to climate forcing or what is referred to as global warming.

Climate change and weather variability are among the biggest challenges to human development as they present a combination of risks that negatively impact human health, global food security, economic development and the natural environment on which much of the human livelihoods depends (Zakarya et al. 2015). Therefore, combating climate change and its impacts is at the core of the 17 SDGs agenda. This is on account that several of the targets under climate change action overlap and impinge on other SDGs. Therefore, risk reduction is advocated in addressing disaster risk drivers, such as poor land management, unsustainable use of natural resources and declining ecosystems, in climate change action and pursuit of SDGs (UN 2015).

### Adaptive capacity

Maintaining and improving livelihoods is one of the farmers’ objectives. These require that farmers adapt to changing policy contexts and environment in which they operate. In this context, adaptation is an active decision-making process framed by risk attitude and perception. The risk attitude and perception is influenced both by farmer characteristics and external factors (Maredia & Minde 2002). The role of adaptive capacity, adaptation process and their implication on use of GHG emissions as an indicator in sustainability metrics is particularly relevant in this context. Such reality creates the need to reassess traditional policy instruments in terms of their adaptability to better reflect climate-related externalities of production and consumption (Banuri & Opschoor 2007).

Coping capacity defines the ability of people, organisations and systems to face and manage adverse conditions, emergencies and disasters using available skills and resources (UNISDR 2015). Coping strategies are short-term responses to a specific shock such as drought, while adaptive strategies entail long-term change in behaviour patterns as a result of shock or stress (Krantz 2001). The capacity to cope requires continuous awareness, resources and good management both in normal times and during adverse conditions (UNISDR 2015). Coping capacities may thus contribute to reduction of disaster risks. However, some coping strategies such as the use of MS as dairy cattle feed without supplementation may be ecologically undesirable (Volenzo 2015).

Reducing farmers’ vulnerability in terms of exposure to risks associated with climate change increases their propensity to engage in more productive economic activities (Siegel 2005). However, high adaptive capacity does not necessarily translate into adaptation actions that reduce vulnerability (IPCC 2007; Moser & Ekstrom 2010). Differences in knowledge, attitudes, beliefs and value system could account for this conclusion. The observation suggests that other interventions, such as advocacy, sustained publicity and education, are necessary ingredients in spurring sustainability in climate change action.

The challenging task in planning adaptation activities is finding ways to combine different measures in a meaningful way to avoid maladaptation. The most attractive adaptation measures are those that offer benefits in the near future and reduce vulnerabilities in the long-term (Mimura et al. 2014). This includes the use of integrated adaptation–mitigation–sustainable development (AMSD) frameworks. As an integral part of wider development goals in transition to sustainability, AMSD frameworks are critical in policy formulation, decision-making, governance and behaviour development (Bizikova, Robinson & Cohen 2007). Adaptation–mitigation–sustainable development planning frameworks include the creation of local implementation pathways that increase opportunities for social learning processes and capacities for effective adaptation and mitigation (FAO 2010). Crop–dairy integration as one of the potential applications from AMSD frameworks, alongside nutrient recycling in MS-based adaptation strategies, invariably reduces methane emission risks and increases carbon sinks, as adaptation co-benefits. This has the potential of contributing to productive and resilient agricultural production systems (Volenzo 2015).

### Disaster risk reduction

Disaster risk reduction is the development and application of policies, strategies and practices designed to minimise vulnerabilities and impacts of disasters through a combination of technical measures to reduce physical hazards and enhance social and economic capacity to adapt (UNISDR 2015). Disaster risk reduction is thus a cost-effective investment in preventing future losses and can be addressed within the context of sustainable development and poverty reduction by integrating risk considerations into policies, plans and programmes (UNISDR 2015). Risk reduction planning process involves the knowledge of situations, processes and systems. In dairy production, mitigation of GHG from feeding strategies reduces the vulnerability of livelihoods from enhanced disaster risks associated with methane emissions. However, methane emission risks in dairy cattle feeds adaptation have been accorded low attention.

### Sustainable livelihoods

Livelihood refers to activities done by a farmer for earning a living. A livelihood is sustainable when it can cope with and recover from stresses and shocks (such as droughts) while maintaining or enhancing its capacities and assets and at the same time not undermining the natural resource base at local and global levels in the short and long-term (Chambers & Conway 1992). Particular livelihood activities by the farmers reflect an explicit (or implicit) multidimensional objective function, including sociocultural and environmental outcomes (Carney 1999). In the context of the above definition, livelihood for a dairy farmer would refer to the well-being of the farmer, in terms of production and sale of milk and milk products, food security, profit margin, survival of the animal asset, employment opportunities as well as contribution to farm productivity and environmental quality (Volenzo 2015).

Overall efficiency, resilience, adaptive capacity and mitigation potential of production systems can be inferred to and improved through its various components, such as soil nutrient management (FAO 2010). At farm level, improved soil fertility management, integrated nutrient management, agroforestry and integrated livestock management lower the negative impacts of farming on natural resources and the environment (UNEP 2012). In this article, sustainable adaptation to climate change is used to refer to management decisions and/or technical measures (newly adopted or intensification of the existing ones) that mitigate GHG emission risks. In a dairy enterprise, feed resources including the purchased components, their quality and availability are among the key determinants of enterprise profitability (Uddin et al. 2010). This also has an impact on productivity and methane emission risks.

The above analysis suggests that the cost of feeds and feeding is important in farmers’ decision-making process and choice of risk management strategies. Exploring this linkage is particularly critical with the rising need for inclusion of climate-related targets in SDGs and a more climate-oriented set of indicators as parts of systems for sustainable development and environmental quality.

### Sustainable adaptation to climate change risks

Definitions of sustainability vary across sectors, but the common theme is to change the way resources are exploited, and how hazards are managed so that adverse impacts downstream or, for subsequent generations, are reduced. Some of the sustainable development indicators pertain to climate change variables, such as level of GHG emissions (Kates, Parris & Leiserowitz 2005). Sustainable principles call for integration of economic and development policies so that in case of conflict between the two, ecological interests are given a preference (UNEP 2012).

The use of MS, different supplementation regimes and their effect on methane emissions can be used in exploring and extending sustainability concept to emission and mitigation of GHGs. In dairy production, energy and protein supplementation reduces methane emissions at herd level (Mills et al. 2003). Thus, supplementation mitigates the negative externalities imposed through CH_4_ emissions and the resultant radiative forcing risks. However, the domino effect of price and production risks in smallholder agriculture with respect to GHG emissions has received little attention. This linkage could be important in the assessment of poverty–maladaptation–environmental degradation nexus.

The dairy sub-sector accounts for about 7% of Kenya’s GDP and 17% of agricultural gross domestic product, in addition to supplying domestic requirements for meat and dairy products (ASDS 2009). Dairy enterprise is one of the few agricultural systems that produce a consistent cash flow over most of the year (Omore et al. 1999), with small-scale farmers accounting for 80% of the total milk production and 70% of the total marketed milk in the country (ASDS 2009). The sub-sector employs about 500 000 people directly and 10 million people indirectly (ASDS 2009). The sub-sector has grown with annual milk production rising from 2.1 billion litres in 2000 to 5.1 billion litres (valued at KES 100 billion) in 2008 (ASDS 2009).

About 40% of dairy cattle in Kenya are found in semi-intensive farming systems (ILRI 2009). This feature, coupled with information that these production systems have the highest maize densities, indicates a high potential for benefits from maize–livestock integration (ILRI 2009). The changing climatic conditions, particularly rainfall and temperature patterns, portend adverse impacts on the agricultural sector and other socio-economic sectors in Kenya. Current projections indicate that such impacts will worsen in the future if significant reductions in the anthropogenic GHGs emissions that are responsible for climate change are not made (GOK 2010).

### Linkages between poverty, environmental degradation and risk management in agriculture

Risk is a combination of the probability of occurrence of an event such as drought and resultant negative consequences such as reduced revenues (Mimura et al. 2014). According to Ullah et al. (2016), there are five major types of risk in agriculture: production or technical risk, market or price risk, legal risk, social risk and human sources of risk. The multiple risks are intertwined and exacerbated by climate change (Bharwani et al. 2005).

Dairy production operations occur in an environment of intertwined risks. Risk refers to any factor that could lower profits or increase expenses, adversely impacting the economic performance of the dairy enterprise (Bailey 2001). Risk occurs from the interaction of vulnerability, exposure and hazard (Mimura et al. 2014). There are many sources of risk in dairy production. These include prices of milk, feeds, crop forage and their production levels, which are influenced by extreme weather variation.

Risk management depends on endowment (Satya 2010; Williams, Hiernaux & Fernandez-Rivera 2000). A household’s portfolio of assets influences farmers’ risk attitude and their ability to respond to risk (Siegel 2005). Accumulated assets allow for more innovative strategies to be pursued with impacts that promote sustainability (Bharwani et al. 2005). Assets also determine the type of activities that can be undertaken. More productive activities (such as high yielding dairy cows) are typically associated with greater risk. Implicitly, assets or resource endowments and their utilisation impact productivity in terms of risk attitude and their effect on expected income and variability of income. Table 2 provides household socio-economic characteristics of the study area, including feed use and supplementation levels as well as mean area under fodder production.

**TABLE 2:** Mean values of household socio-economic characteristics in Kakamega and Bungoma counties.

Household characteristic	Maize zone (*N* = 221)	Sugarcane zone (*N* = 179)	Mean for both (*N* = 400)	2-tailed ^χ2^ *p*
Off-farm income	35 000	25 000	30 000 ± 5000	0.0500*
Crop income (Kes)/Yr	75 000	50 000	62 500 ± 12 500	0.0546*
Dairy income (Kes)/Yr	45 000	17 500	31 500 ± 5450	0.0010**
% using biogas	2	5	3.5 ± 0.5	0.0515*
%Grain supplementation	20	5	22.5 ± 1.2	0.0010**
%Energy supplementation	15	5	10 ± 1.1	0.0010**
%Protein supplementation	8	5	6.5 ± 1.5	0.7340
Livestock Unit (LU)	4.45	2.85	3.65 ± 0.05	0.0510*
Acreage Napier (acres)	0.1	0.2	0.15 ± 0.1	0.0745
Land size (acres)	3.5	2.5	3 ± 1.2	0.05330*
Milk production/day/cow (Litres)	4.2	1.8	3 ± 1.5	0.05120*
% SCT use	15	78	46.5 ± 5.3	0.0010**
% Stover use	95	65	80 ± 2.5	0.0010**

* , Significant difference at 0.05 and 0.01 level of significance.

** , Significant difference at 0.05 and 0.01 level of significance.

A farmer may perceive a technology as high risk if it requires investing a higher proportion of his or her limited resources (such as cash for subsistence small-scale farming or improving knowledge and management skills of a farmer) or foregoing a practice that is culturally valued (such as well adapted but low external input-dependent local cattle breeds) in the current system (Maredia & Minde 2002). This could explain about the low to non-existent supplementation of MS in the maize–sugarcane belt of western Kenya (Volenzo 2015). The low energy supplementation to non-existent supplementation of MS as a risk management strategy not only reduces income but also increases CH_4_ emission risks per unit production. The low levels of energy (10 ± 1.1%) and protein (6.5 ± 1.5%) supplementation by households is reflected in low milk production levels (3 ± 1.5 L) against potential of 20 L/day for dairy breeds of medium milk production potential in the study area. As shown in Table 1, low energy and protein supplementation increase methane emission risks.

In the above analysis, accumulated capital allows for purchase and use of external inputs, such as dairy feed supplements. This can significantly impact poverty outcomes and environmental degradation, as external inputs such as energy and protein supplements in dairy production significantly influence methane emission risks that impact environmental sustainability. Higher productivity of the dairy herd from increased use of external inputs not only reduces poverty but also reduces methane emission per unit litre of milk produced.

### Policy implications

The authors have used methane emission levels in the adaptation of dairy feeding strategies as a measure of sustainable development. It is apparent that mitigating GHG emission risks is one of the pillars of sustainable adaptation to climate change in dairy production. In this study, price risks are intricately associated with maladaptation to climate change among resource-poor farmers. While anecdotal evidence points to pollution and/or environmental degradation caused by the use of external inputs in crop agriculture, considerations of GHG emission levels in the use of MS suggest otherwise. Increased levels of energy and protein supplementation mitigate GHG, with positive impacts on environmental and financial sustainability. The counterfactual seems to suggest that any policy on adaptation and poverty–environmental degradation nexus has to be resource specific. From the analysis, it is clear that climate change can be a driver of disaster risks when economic vulnerabilities that reduce access to inputs and resources that mitigate GHGs are prevalent. Tackling the underlying disaster risk drivers, such as cognitive failure, poverty and poor natural resource management, is thus critical to risk reduction.

## Conclusion

Using MS as a specific feed resource in price and production risk contexts, this study identified potential pathways on poverty–production risks–maladaptation to climate change–Environmental degradation nexus. Simulation of methane emissions from small-scale farmers’ dairy cattle feeding adaptation strategies suggests that low or non-existent supplementation in MS-based rations is associated with higher-than-average methane emissions. The findings of the study underscore the centrality of hazard vulnerability risk assessment and multisectorial planning in the design of sustainable adaptation frameworks. Accordingly, adaptation frameworks should pay close attention to socio-economic issues, social organisations and institutions as the basis for risk-informed policies in general and for the assessment, prioritisation, monitoring and evaluation of climate change adaptation actions for the agricultural sector in particular.
